# Penicillin allergy de-labelling by non-allergists: a comparison of testing protocols

**DOI:** 10.1093/jacamr/dlad134

**Published:** 2023-12-19

**Authors:** Neil Powell, Shuayb Elkhalifa, Jonathan Sandoe

**Affiliations:** School of Biomedical Sciences, University of Plymouth, Plymouth, UK; Pharmacy Department, Royal Cornwall Hospital Trust, Truro, UK; Allergy and Immunology Department, Respiratory Institute, Cleveland Clinic Abu Dhabi, Abu Dhabi, United Arab Emirates; Leeds Institute of Medical Research, University of Leeds, Leeds, UK

## Abstract

Optimizing penicillin allergy de-labelling (PADL) to ensure patients with an incorrect penicillin allergy record are de-labelled with minimal patient harm is important for antibiotic stewardship. The heterogeneity of inclusion and exclusion criteria in the published penicillin allergy testing protocols risks suboptimal delivery of PADL. We compared the similarities and the differences between non-allergist-delivered PADL testing protocols and make suggestions for harmonization.

The observed variation in testing practice has two broad elements: (i) definitions and terminology; and (ii) differences in the acceptability of perceived risk. All direct drug provocation testing (DDPT) protocols included patients with benign delayed rash as eligible for testing, although the remoteness of the rash, and the terminology used to describe the rash, differed. Patients with features of potential IgE reactions were excluded from most DDPT protocols, but not all of them. There was differing advice on how to manage patients who had subsequently tolerated penicillin since the index reaction and differences in which patients were considered ineligible for DDPT due to acuity of illness, comorbidities and concomitant medications.

Standardization of the terminology used in penicillin allergy testing protocols and consensus on inclusion and exclusion criteria are required for safe and efficient PADL delivery at scale by non-allergists.

## Introduction

Penicillin allergy records are common, often incorrect, and associated with patient, healthcare system and wider societal harm.^1^ Several societies have produced guidelines and toolkits for non-allergists to practise safe de-labelling of patients with low-risk penicillin allergy records.^2–4^ The testing criteria differ between these guidelines and therefore the testing strategies in the literature also differ.^5^

Optimizing penicillin allergy de-labelling (PADL) to ensure the greatest proportion of patients with a penicillin allergy are de-labelled with minimal patient harm is important for antibiotic stewardship programmes. There are now several validated penicillin allergy testing decision-support tools in the literature to help clinicians deliver PADL safely but there is variation in the accepted levels of risk.^6–10^ Our recent systematic review identified 69 studies reporting the safe delivery of PADL by non-allergists.^5^ Studies used either a single testing method or a combination of testing methods, which included de-label on history alone (or direct de-label, DDL), de-label after a direct drug provocation test (DDPT) or skin testing followed by provocation testing (ST/PT). There was heterogeneity in the testing protocol inclusion and exclusion criteria between studies.^5^

We set out to compare the similarities and the differences between the DDL testing protocols and the DDPT protocols and to make suggestions on how protocols might be harmonized to optimize PADL.

## Methods

Studies that fulfilled the criteria to be included in a recent systematic review were eligible.^5^ Studies that used DDL and/or DDPT were identified and included if they provided information on inclusion and exclusion criteria for penicillin allergy assessment and testing methods. Patient allergy symptoms and patient factors that included and excluded patients from both DDL and DDPT methods were then extracted from the original publications, collated in a matrix and compared.

## Results

Sixteen studies using DDPT^4,8,11–24^ and 15 using DDL^13–17,19,21,25–32^ were included with some using more than one method.

### Inclusion criteria for DDPT: allergy history reported symptoms (Tables 1 and 2)

#### Allergy disproven or no evidence of personal reaction to penicillin

Four studies offered DDPT to patients with penicillin allergy in their health record, but upon questioning, the patient denied a penicillin allergy. One study offered DDPT to patients with a family history of penicillin allergy only, or avoidance of penicillin due to fear. In the remaining 11 studies this patient group was not defined. Of note, patients avoiding penicillin due to fear didn’t appear in the DDL protocols of any of the studies that used both DDPT and DDL methods.

**Table 1.** DDPT method inclusion criteria for testing: allergy history factors

		Ethnicity of participants recorded	Non-white participants (%)	Allergy disproven or no evidence of personal reaction to penicillin				Type A reactions					Unknown reactions				
Reference	PADL methods used in the study			Known tolerance of a penicillin since the original reaction occurred	Avoidant from fear of allergy only	Family history of penicillin allergy only	Records of allergy but patient denied allergy	Nausea, vomiting or diarrhoea	Type A reaction (non-immune adverse drug reaction, such as nausea or vomiting)	Headache	Thrush	Other symptoms, non-allergy	Remote, unknown reaction (patient cannot recall)	Unknown reaction >10 years ago or date can’t be recalled	Unknown reaction >10 years ago	Unknown symptoms occurring ≥6 months ago and ≥1 h after drug administration	Remote childhood reaction with limited details
Blumenthal *et al.*, 2019^11^	DOC/DIVC	No	—				✓										
Blumenthal *et al.*, 2015^12^	DOC	No	—				✓										
Chua *et al.*, 2020^13^	DDL, DOC	yes	8.2												✓		
Devchand *et al.*,2019^14^	DDL, DOC, ST/OC	No	—												✓		
du Plessis *et al.*, 2019^15^	DDL, DOC	Yes	54														
Ham *et al.*, 2021^16^	DDL, DOC, ST/OC	No	—														
Harper *et al.*, 2021^17^	DDL, DOC, ST, ST/OC	No	—										✓				
Lin *et al.*, 2020^18^	DOC	No	—					✓					✓				
Livirya *et al.*, 2020^19^	DDL, DOC	No	—													✓	
Maguire *et al.*, 2020^20^	DOC	No	—				✓										
Sacco *et al.*, 2019^21^	DDL, DOC/DIVC	No	—				✓										
Savic *et al.*, 2019^22^	DOC	No	—					✓			✓						
Sneddon *et al.*, 2021^4^	DOC	No	—					✓							✓		
Steenvoorden *et al.*, 2021^23^	DOC	No	—					✓		✓							
Stone *et al.*, 2020^8^	DOC	yes	9	✓	✓	✓		✓				✓	✓				✓
Trubiano *et al.*, 2018^24^	DOC	No	—						✓								

DOC, direct oral challenge; DIVC; direct IV challenge; ST/OC, skin test followed by oral challenge.

**Table 2.** DDPT method inclusion criteria for testing: allergy history factors

Phenotype group		Delayed non-severe skin rashes												Potential IgE-mediated reactions	
Reference	PADL methods used in the study	Delayed onset rash	Non-itchy rash	Benign rash	Minor rash only	Maculopapular rash >10 years ago	Maculopapular rash only	Delayed (>2 h) maculopapular rash	Self-limited cutaneous rash at any point	Limited cutaneous reaction (including rash and hives)	Childhood exanthem only	Rash, rhinitis, GI symptoms if >12 months ago	Diffuse rash or localized swelling >10 years ago	IgE- mediated, immediate (<2 h) if >10 years ago	Immediate symptom onset (<6 h) if only 1–2 of the symptoms in the mild/moderate/severe categories and >10 years ago.
Blumenthal *et al*., 2019^11^	DOC/DIVC				✓		✓								
Blumenthal *et al*., 2015^12^	DOC				✓		✓								
Chua *et al*., 2020^13^	DDL, DOC					✓					✓		✓		
Devchand *et al*., 2019^14^	DDL, DOC, ST/OC					✓					✓		✓		
du Plessis *et al*., 2019^15^	DDL, DOC	✓													
Ham *et al*., 2021^16^	DDL, DOC, ST/OC				✓			✓						✓	
Harper *et al*., 2021^17^	DDL, DOC, ST, ST/OC				✓										
Lin *et al*., 2020^18^	DOC											✓			✓
Livirya *et al*., 2020^19^	DDL, DOC									✓					
Maguire *et al*., 2020^20^	DOC				✓		✓								
Sacco *et al*., 2019^21^	DDL, DOC/DIVC				✓		✓								
Savic *et al*., 2019^22^	DOC		✓												
Sneddon *et al*., 2021^4^	DOC	implied through exclusion criteria													
Steenvoorden *et al*., 2021^23^	DOC			✓											
Stone *et al*., 2020^8^	DOC								✓						
Trubiano *et al*., 2018^24^	DOC			✓		✓					✓				
Protocols with phenotype group, *n*/*N* (%)		16/16 (100)													2/16 (12.5)

DOC, direct oral challenge; DIVC; direct IV challenge; ST/OC, skin test followed by oral challenge.

One study offered DDPT to patients who had tolerated a penicillin since index reaction but without specifying whether the tolerance needed to be to a specific named penicillin or whether known tolerance could be to any of the penicillin antibiotics.

#### Non immune-mediated side effects/intolerances

Studies that de-labelled using both DDL and DDPT did not include patients with an intolerance in their DDPT protocols. Of the nine studies that only offered DDPT, six (67%) included intolerances. The three studies that didn’t include intolerances did not describe how this patient group were managed and were based on the same testing protocol.^12^

#### Delayed non-severe skin rashes

All 16 studies that de-labelled using DDPT included patients with delayed non-severe skin reactions. The description of the rash varied between studies. Descriptions of rashes that met inclusion criteria for DDPT included ‘maculopapular rash’, ‘benign rash’, ‘non-itchy rash’, ‘self-limiting rash’, ‘limited cutaneous reaction which includes hives’ and ‘childhood exanthem’. Three studies stipulated the rash needed to have occurred >10 years ago, one included patients if the rash occurred >1 year ago, and the remainder did not stipulate how remote the delayed rash needed to be to meet DDPT inclusion criteria. One study defined delayed as occurring >2 h post exposure to penicillin.

#### Unknown reactions

In 9 of 16 studies (56%), unknown reactions were an inclusion criterion for DDPT. There was variation in time elapsed since index unknown reaction from >6 months to >10 years ago and ‘childhood’. The study that included unknown reactions >6 months ago also required the onset of the reaction to be ≥1 h after drug administration. Of the remaining seven protocols, five explicitly excluded unknown reactions from DDPT and two did not mention unknown reactions.

#### Potential IgE-mediated reactions

Remote potential IgE-mediated reactions were included in 3 of 16 (19%) DDPT protocols, comprising IgE-mediated reactions >10 years ago,^16,18^ and urticaria if a sole symptom >5 years ago (1 study).^8^

### Allergy history exclusion criteria for DDPT (Tables 3 and 4)

#### Severe delayed reactions

All 16 studies excluded Gell and Coombes (G&C) type 4 reactions, of which 10 also excluded G&C type 2 and 3 reactions. Of the remaining six studies, two excluded symptoms consistent with type 2 and 3 reactions and four studies did not identify type 2 and 3 reactions as exclusion criteria.^4,15,18,22^

**Table 3.** DDPT method exclusion criteria for testing: allergy history factors

		Potential type 1 reactions													Broader G&C defined allergy groups	
Reference	PADL methods used in the study	IgE- mediated, immediate (<2 h) if <10 years ago	Type 1	Reaction definitely occurred within 1 h	Anaphylaxis	Angioedema	Swelling of face/body	Disseminated hives/urticaria/flushing/pruritis	Respiratory symptoms	Collapse or dizziness	Shock	Weak pulse	Loss of consciousness	Itchy rash	Type 1–4 G&C	G&C types 2–4
Blumenthal *et al.*, 2019^11^	DOC/DIVC														✓	
Blumenthal *et al.*, 2015^12^	DOC														✓	
Chua *et al.*, 2020^13^	DDL, DOC														✓	
Devchand *et al.*, 2019^14^	DDL, DOC, ST/OC														✓	
du Plessis *et al.*, 2019^15^	DDL, DOC		✓													
Ham *et al.*, 2021^16^	DDL, DOC, ST/OC	✓														✓
Harper *et al.*, 2021^17^	DDL, DOC, ST, ST/OC														✓	
^a^Lin *et al.*, 2020^18^	DOC	complicated algorithm														
Livirya *et al.*, 2020^19^	DDL, DOC				✓	✓			✓							
Maguire *et al.*, 2020^20^	DOC														✓	
Sacco *et al.*, 2019^21^	DDL, DOC/DIVC														✓	
Savic *et al.*, 2019^22^	DOC				✓	✓	✓		✓	✓				✓		
Sneddon *et al.*, 2021^4^	DOC		✓													
Steenvoorden *et al.*, 2021^23^	DOC														✓	
Stone *et al.*, 2020^8^	DOC				✓	✓	✓	✓	✓		✓	✓	✓		✓	
Trubiano *et al.*, 2018^24^	DOC				✓	✓										
Protocols with phenotype group, *n*/*N* (%)		8/16 (50)													10/16 (62.5)	

DOC, direct oral challenge; DIVC; direct IV challenge; ST/OC, skin test followed by oral challenge.

^a^Lin—immediate symptom onset (<6 h) if only >2 of the symptoms in the mild/moderate/severe categories [mild—rash, rhinitis, GI symptoms; moderate—urticaria, oedema, mild dyspnoea, fever, indication for hospitalization and absence of the criteria mentioned under severe; severe—angioedema, stridor, severe dyspnoea, haemodynamic instability, (nor)epinephrine use, severe skin reactions (Stevens-Johnson Syndrome, toxic epidermal necrolysis), admission to monitoring ward].

**Table 4.** DDPT method exclusion criteria for testing: allergy history factors

		Bone, liver, kidneys		Severe delayed skin reactions				Unknown		Timing since reaction			Other		Required treatment	
Reference	PADL methods used in the study	Interstitial nephritis (t3), vasculitis (t2), haemolytic anaemia (t2)	β-Lactam-associated renal impairment (t3) or liver injury (t3)	Type 4 reactions (SJS/TEN, DRESS, AGEP)	Drug-induced exfoliative dermatitis	Severe blistering rash	Generalized cutaneous reaction	Vague history/inconclusive interview	Unknown reaction	Hypersensitivity reaction within the last 5 years	Index allergic reaction was <4 weeks ago	Reaction <15 years ago	Systemic symptoms	Severe GI symptoms (diarrhoea, vomiting)	Required urgent medical care	Symptoms required hospital treatment
Blumenthal *et al.*, 2019^11^	DOC/DIVC								✓							
Blumenthal *et al.*, 2015^12^	DOC								✓							
Chua *et al.*, 2020^13^	DDL, DOC															
Devchand *et al.*, 2019^14^	DDL, DOC, ST/OC															
du Plessis *et al.*, 2019^15^	DDL, DOC			✓				✓		✓	✓		✓			
Ham *et al.*, 2021^16^	DDL, DOC, ST/OC															
Harper *et al.*, 2021^17^	DDL, DOC, ST, ST/OC															
Lin *et al.*, 2020^18^	DOC			✓												
Livirya *et al.*, 2020^19^	DDL, DOC	✓		✓	✓	✓	✓									
Maguire *et al.*, 2020^20^	DOC								✓							
Sacco *et al.*, 2019^21^	DDL, DOC/DIVC								✓							
Savic *et al.*, 2019^22^	DOC					✓						✓				✓
Sneddon *et al.*, 2021^4^	DOC			✓											✓	✓
Steenvoorden *et al.*, 2021^23^	DOC															
Stone *et al.*, 2020^8^	DOC													✓		
Trubiano *et al.*, 2018^24^	DOC		✓	✓												

DOC, direct oral challenge; DIVC; direct IV challenge; ST/OC, skin test followed by oral challenge; SJS/TEN, Stevens-Johnson syndrome/toxic epidermal necrolysis; DRESS, drug reaction with eosinophilia and systemic symptoms; AGEP, acute generalized exanthematous pustulosis.

#### Type 1 immediate reactions

Eleven studies excluded type 1 allergies,^4,8,11–15,17,20,21,23^ three excluded patients with symptoms consistent with severe type 1 allergies such as history of collapse, angioedema and anaphylaxis,^19,22,24^ and two studies only excluded severe IgE-mediated reactions if they occurred less than 10 years ago.^16,18^

#### Other severe features

One study excluded patients if there were systemic symptoms, and one if the patient had severe gastrointestinal (GI) symptoms; requiring urgent medical care was an exclusion criterion in one study, and requiring hospital treatment was an exclusion criterion in two studies.

### Patient factor exclusion criteria for DDPT (Tables 5 and 6)

#### Concurrent medication

Four studies excluded patients taking beta-blockers, one withheld beta-blockers, if possible, and one explicitly stated that patients taking beta-blockers were not excluded. The remainder (63%) did not mention beta-blockers. Three studies excluded patients on angiotensin-converting enzyme inhibitors (ACEi), the remainder did not mention ACEi (81%). Four studies excluded patients taking antihistamines and one excluded these patients only if a histamine skin test was negative. The remaining 11 (69%) studies did not mention antihistamines as an exclusion criterion. Corticosteroid use was an exclusion criterion in three studies: if doses were >20 mg/day in one study; >10 mg/day in another study; and one study did not specify a dose. The remainder (81%) did not mention corticosteroids.

**Table 5.** DDPT method exclusion criteria for allergy testing: patient factors

		Other medication							Acuity of illness							
Reference	PADL methods used in the study	On beta-blocker	On ACEi	Antihistamines	Antihistamines within 24 h	Corticosteroids (>20 mg pred/day)	Steroids no dose given	Corticosteroids (>10 mg pred/day)	ICU admission (current episode)	In ICU	Medical emergency team call within last 24 h	Critically unwell	Actively dying	Haemodynamic instability	Systolic blood pressure <100 mmHg	Medically unstable, NEWS ≥2
Blumenthal *et al.*, 2019^11^	DOC/DIVC	✓	✓													
Blumenthal *et al.*, 2015^12^	DOC	✓	✓													
Chua *et al.*, 2020^13^	DDL, DOC							✓	relative CI			✓		✓		
Devchand *et al.*, 2019^14^	DDL, DOC, ST/OC									✓						
du Plessis *et al.*, 2019^15^	DDL, DOC	✓	✓											✓	✓	
Ham *et al.*, 2021^16^	DDL, DOC, ST/OC	× (not exclusion)		✓^a^								relative CI		✓		
Harper *et al.*, 2021^17^	DDL, DOC, ST, ST/OC			✓	✓											
Lin *et al.*, 2020^18^	DOC															
Livirya *et al.*, 2020^19^	DDL, DOC					✓							✓	✓		
Maguire *et al.*, 2020^20^	DOC	held if possible												✓		
Sacco *et al.*, 2019^21^	DDL, DOC/DIVC															
Savic *et al.*, 2019^22^	DOC			✓			✓									
Sneddon *et al.*, 2021^4^	DOC	✓		✓												✓
Steenvoorden *et al.*, 2021^23^	DOC											✓				
Stone *et al.*, 2020^8^	DOC													✓		
Trubiano *et al.*, 2018^24^	DOC										✓			✓		

DOC, direct oral challenge; DIVC; direct IV challenge; ST/OC, skin test followed by oral challenge.

^a^If histamine ST negative.

**Table 6.** DDPT method exclusion criteria for allergy testing: patient factors

		Pregnancy/breastfeeding		Cognitive impairment		Propensity to be allergic		Age/other		Respiratory disease				Heart disease			Immunosuppression	
Reference	PADL methods used in the study	Pregnancy	Breastfeeding	Allergy unavailable due to due patient cognitive impairment and no collateral history	Cognitive impairment	History of idiopathic urticaria or anaphylaxis	Severe anaphylaxis of any cause in the last 4 months	Unable to consent	Age >70 years	Patients with asthma and/or COPD that is (i) poorly controlled and/or (ii) required hospitalization within 1 year of consent and/or (iii) ever admitted to an ICU and/or (iv) FEV1, 40%	Moderate to severe pulmonary disease	Uncontrolled asthma	Unstable asthma (steroids in the last 6 months)	Patients with significant cardiovascular disease (active angina; arrhythmias; NSTEMI or CABG within 6 months)	Moderate to severe cardiac disease	Unstable coronary artery disease	Immunosuppression	Immunosuppressive drugs
Blumenthal *et al.*, 2019^11^	DOC/DIVC																	
Blumenthal *et al.*, 2015^12^	DOC																	
Chua *et al.*, 2020^13^	DDL, DOC	✓		✓														
Devchand *et al.*, 2019^14^	DDL, DOC, ST/OC				✓													
du Plessis *et al.*, 2019^15^	DDL, DOC	✓						✓	✓	✓				✓				
Ham *et al.*, 2021^16^	DDL, DOC, ST/OC	✓									✓				✓			
Harper *et al.*, 2021^17^	DDL, DOC, ST, ST/OC																✓	✓
Lin *et al.*, 2020^18^	DOC																	
Livirya *et al.*, 2020^19^	DDL, DOC	✓															✓	
Maguire *et al.*, 2020^20^	DOC																	
Sacco *et al.*, 2019^21^	DDL, DOC/DIVC																	
Savic *et al.*, 2019^22^	DOC	✓	✓										✓					
Sneddon *et al.*, 2021^4^	DOC	✓										✓				✓		
Steenvoorden *et al.*, 2021^23^	DOC						✓											
Stone *et al.*, 2020^8^	DOC			✓														
Trubiano *et al.*, 2018^24^	DOC	✓		✓		✓												

DOC, direct oral challenge; DIVC; direct IV challenge; ST/OC, skin test followed by oral challenge FEV1, forced expiratory volume in 1 s; NSTEMI, non-ST-elevation myocardial infarction; CABG, coronary artery bypass graft.

#### Comorbidities and acuity of illness

Six studies did not exclude patients due to high acuity of illness; 10 did. Seven excluded patients that were haemodynamically unstable, two excluded patients currently in the ICU or had been in the ICU, one if the patient was critically unwell and one if the New Early Warning Score (NEWS) score was ≥2.^33^ Cognitive impairment or inability to give consent were exclusion criteria in five studies. Four studies excluded patients with respiratory disease and three studies excluded those with heart disease. Immunosuppressed patients were excluded in two studies, pregnancy was excluded in seven studies and breastfeeding in one study.

### De-label on history alone (Tables 7 and 8)

The inclusion criteria were poorly defined in 4 of the 15 studies that used DDL.

**Table 7.** De-label on history alone (DDL) when there has been subsequent tolerance of penicillin since the index reaction

		Previous penicillin tolerance				
Reference	PADL method used	Not defined	Type 1 history with recent use of penicillin (type of pen not defined)	Previously tolerated any penicillin (regardless of penicillin allergy risk group)	Subsequent tolerance to the implicated penicillin	Low risk (i.e. DOC candidate) and tolerated penicillin antibiotics since the index reaction
Chua *et al.*, 2020^13^	DDL, DOC				✓	
Devchand *et al.*, 2019^14^	DDL, DOC, ST/OC					
du Plessis *et al.*, 2019^15^	DDL, DOC			✓		
Gaudreau *et al.*, 2021^25^	DDL, ST/OC	✓				
Griffith *et al.*, 2020^26^	DDL, ST/(±)OC	✓				
Ham *et al.*, 2021^16^	DDL, DOC, ST/OC			✓		
Harper *et al.*, 2021^17^	DDL, DOC, ST, ST/OC		✓			
Jones *et al.*, 2019^27^	DDL, ST/(±)OC	✓				
Livirya *et al.*, 2020^19^	DDL, DOC					✓
Mitchell *et al.*, 2021^28^	DDL	✓				
Sacco *et al.*, 2019^21^	DDL, DOC/DIVC			✓		
Sigona *et al.*, 2016^32^	DDL			✓		
Song *et al.*, 2021^29^	DDL					
Wall *et al.*, 2004^31^	DDL, ST/OC		✓			
Taremi *et al.*, 2019^30^	DDL, ST/OC					

DOC, direct oral challenge; DIVC; direct IV challenge; ST/OC, skin test followed by oral challenge.

**Table 8.** De-label on history alone (DDL): history factors

		Intolerances									
Reference	Penicillin allergy de-label method used	GI symptoms (nausea, vomiting, diarrhoea)	Mild adverse drug reaction such as GI upset	Mild neurological manifestation (headache, depression, mood disorder)	Chills	Headache	Fatigue	Intolerance (not defined)	Non-allergy adverse event such as nausea, vomiting or headaches	Mild renal impairment	Mild hepatic enzyme derangement
Chua *et al.*, 2020^13^	DDL, DOC	✓		✓						✓	✓
Devchand *et al.*, 2019^14^	DDL, DOC, ST/OC	✓		✓						✓	✓
du Plessis *et al.*, 2019^15^	DDL, DOC								✓		
Gaudreau *et al.*, 2021^25^	DDL, ST/OC										
Griffith *et al.*, 2020^26^	DDL, ST/(±)OC										
Ham *et al.*, 2021^16^	DDL, DOC, ST/OC										
Harper *et al.*, 2021^17^	DDL, DOC, ST, ST/OC										
Jones *et al.*, 2019^27^	DDL, ST/(±)OC										
Livirya *et al.*, 2020^19^	DDL, DOC		✓								
Mitchell *et al.*, 2021^28^	DDL										
Sacco *et al.*, 2019^21^	DDL, DOC/DIVC										
Sigona *et al.*, 2016^32^	DDL										
Song *et al.*, 2021^29^	DDL	✓			✓	✓	✓				
Wall *et al.*, 2004^31^	DDL, ST/OC										
Taremi *et al.*, 2019^30^	DDL, ST/OC							✓			

DOC, direct oral challenge; DIVC; direct IV challenge; ST/OC, skin test followed by oral challenge.

#### Tolerance of penicillin since index reaction

Eight (53%) studies recommended de-labelling on history alone if there was evidence of tolerance to penicillin since the index reaction; four studies recommended de-label regardless of previous allergy history, two recommended de-label if history was of type 1 allergy and subsequent penicillin tolerance (name of penicillin not specified), one recommended de-label if subsequent tolerance to the implicated penicillin and one recommend de-label if the patient had a low-risk allergy history and tolerance to any penicillin since index reaction.

#### History of intolerance/type A reactions

Six (67%) studies recommended DDL if the patient had experienced an intolerance only. These included GI symptoms in five studies, mild neurological manifestations in three studies, mild renal or hepatic derangement in two studies, and with one each including intolerance (non-defined), chills and fatigue.

## Discussion

Although there are similarities with the inclusion and exclusion criteria used in non-allergist penicillin allergy de-labelling testing protocols, there is marked heterogeneity. It appears that the observed variation in testing practice has two broad elements: (i) definitions and terminology used; and (ii) differences in the acceptability of perceived risk.

The inclusion of delayed non-severe skin rashes is consistent across all DDPT protocols, but the definitions used for delayed non-severe skin rashes and the time intervals since a reaction varied. Agreement and a standardized definition of which rash phenotypes should be categorized as a delayed non-severe skin rash would potentially enable more patients to be de-labelled.

Three studies did not include penicillin intolerance as an indication for DDPT. It may be that these patients were managed outside the testing protocol. The remaining studies included intolerance as an inclusion criterion for DDPT. GI system intolerance was identified as intolerance in all protocols but there was variation in the inclusion of other symptoms that might be phenotyped as an intolerance, e.g. headache. A comprehensive list of what would constitute intolerance may be useful for non-allergists to confidently determine reactions that might be grouped as an intolerance and therefore optimize the potential candidates for PADL.

There were significant differences concerning the management of patients with unknown reactions with some excluding, and some including, patients with unknown reactions. Of those that included unknown reactions, there were differences in the time elapsed since the index reaction with ‘childhood’, more than 10 years ago, or unknown time lapse all represented. When assessing unknown reactions, no studies reported assessing extent of cognitive decline as a risk when undertaking allergy assessment. International consensus on how to manage patients with unknown reactions and how to manage patients with language barriers to communication and those with differing levels of cognitive impairment would optimize PADL. Of note, the majority of studies were from high-income countries (predominantly USA and Australia) and the study population ethnicity was reported in a minority of studies, and where reported, predominantly in white populations, making transferability of data to low/middle-income and diverse populations challenging.

There were fewer differences in relation to management of potential IgE-mediated reactions. While the vast majority excluded IgE-mediated reactions, two studies included remote urticaria and one included IgE-mediated reactions if >10 years ago. Why some teams tolerate the IgE risk and others do not needs to be understood. PEN-FAST is a validated clinical decision rule that enables point-of-care risk stratification of patients with penicillin allergy.^9^ Using this decision rule, the likelihood of a positive penicillin allergy test in a patient with a history of urticaria more than 5 years ago is assessed as ‘very low’ (<1%) and if the same patient had urticaria less than 5 years ago that risk increases to ‘low’ (5%).^9^ The evidence to date supports inclusion of patients with a history of isolated urticaria in DDPT protocols and this patient group warrants consideration in testing protocols. However, there is emerging evidence suggesting that patients with a history of urticaria exhibiting three specific high-risk features should be approached with caution. These features include reactions manifesting within 1 h of the first dose, and subsiding within 1 day.^34^

All studies excluded severe type 4 reactions and the majority excluded those with types 2 and 3 or specified symptoms consistent with types 2 and 3 but notably four studies did not exclude this group. It seems inconceivable that patients with these reactions would be tested by DDPT and so this is either an oversight or thought to be too rare to explicitly mention in the protocol.

There was inconsistency between studies regarding oral challenge testing while on concomitant medications. Several studies excluded patients for testing if they were taking beta-blockers, antihistamines, ACEi or oral corticosteroids but most of the studies did not make mention of these drugs as an exclusion criterion, with one explicitly stating that beta-blockers were not an exclusion criterion. We believe the rationale for excluding patients on corticosteroids and antihistamines is due to the risk of these medications suppressing mast cell release of histamine and therefore masking IgE-mediated allergic reactions. Beta-blockers might mask the signs of anaphylaxis and might blunt the effect of adrenaline when treating patients for anaphylaxis, although a recent report suggests this not to be the case.^35^ ACEi are associated with bradykinin-induced angioedema, an immunopathological pathway that differs from histamine or mast cell-induced angioedema. ACEi-induced angioedema is uncommon, affecting 0.1%–0.7% of patients taking ACEi, unpredictable and can occur at any time.^36^ Excluding patients taking these common medications reduces the opportunity to de-label inpatients at the point of need and reduces the potential impact of PADL on patient care. Given the low risk of these patients selected for DDPT to have an allergic reaction, the risk–benefit is probably in favour of not excluding these patients. More data and consensus are required.

The majority of studies had an acuity of illness exclusion criterion, but the severity of illness varied between studies. The reasons for excluding patients with high-acuity illness are likely due to the difficulty differentiating some of the severe infection symptoms from severe IgE-mediated symptoms and moreover infections reduce the threshold of developing anaphylactic reactions and so a patient with acute infection also suffering an anaphylactic reaction may result in death when they may have otherwise survived the episode of care.^37^ However, the risk of allergy in patients selected for DDPT is low and comparable to that for the general population. The benefit of PADL to antibiotic stewardship and patient outcomes needs to be weighed against this small risk of allergy,^38^ one that we do not consider in patients who are not labelled as penicillin allergic in the general population who carry similar risks of allergic reaction.

Most studies recommended de-label on history alone if there was evidence of tolerance to a penicillin since the index reaction but there was significant heterogeneity with the advice on index reactions and type of penicillin tolerated. Several studies recommended de-labelling if there had been tolerance to any penicillin, regardless of the previous allergy history, i.e. regardless of whether the patient had a previous IgE-mediated reaction or a lower risk allergy history. Two studies specifically suggested de-labelling of those with a history of type 1 reactions and subsequent tolerance to penicillin (type not specified). IgE reactions are reported to be triggered by R1 penicillin side chains in 25% of allergic reactions and the β-lactam ring in 75% of European studies,^39^ and so there remains a risk that if somebody had an IgE-mediated reaction to the amoxicillin R1 side chain and subsequently tolerated phenoxymethylpenicillin they might be de-labelled but remain at risk of a serious IgE-mediated reaction if exposed to amoxicillin in the future. Only one study specified that the subsequent tolerated penicillin should be the index penicillin and one study suggested de-label on history alone if the patient tolerated any penicillin only if the index reaction was a low-risk allergy history, i.e. one that would be amenable to DDPT. There remains uncertainty as to the safest way to manage this patient group.

De-labelling patients on history alone when the index reaction was an intolerance was not commonly included in DDL protocols and when it was, there was inconsistency with which intolerance could be de-labelled on history alone. What we mean by intolerances needs further definition and which intolerances are amenable to DDL also needs further refinement.

### Conclusions

While there are similarities in approach, there is wide variation in the terminology, in the inclusion and exclusion criteria for DDPT and DDL in the testing protocols reported in the literature. As we gain more confidence with DDPT and DDL and gather further evidence of the safety of DDL and DDPT, particularly non-allergist-delivered interventions, we need to standardize the terminology and reach consensus on inclusion and exclusion criteria to ensure safe and efficient penicillin allergy assessment and to deliver PADL confidently at scale. To achieve global harmonization of PADL, it is imperative that studies meticulously report racial data and prioritize ethnic diversity within their tested populations when validating toolkits.
